# Clinical Outcomes for Previously Treated Patients with Advanced Gastric or Gastroesophageal Junction Cancer: A Systematic Literature Review and Meta-Analysis

**DOI:** 10.1007/s12029-023-00932-5

**Published:** 2023-05-23

**Authors:** Lauren A. Abderhalden, Ping Wu, Mayur M. Amonkar, Brian M. Lang, Sukrut Shah, Fan Jin, Andrew M. Frederickson, Ali Mojebi

**Affiliations:** 1 MSD, Zurich, Switzerland; 2 PRECISIONheor, Vancouver, BC Canada; 3 Merck & Co., Inc, Rahway, NJ USA; 4 PRECISIONheor, New York, NY, USA

**Keywords:** Metastatic gastric cancer, Pretreated gastric cancer, Chemotherapy, Targeted therapy, Systematic literature review, Meta-analysis

## Abstract

**Purpose:**

Although second-line treatments improve survival compared to best supportive care in patients with advanced gastric cancer with disease progression on first-line therapy, prognosis remains poor. A systematic review and meta-analysis were conducted to quantify the efficacy of second-or-later line systemic therapies in this target population.

**Methods:**

A systematic literature review (January 1, 2000 to July 6, 2021) of Embase, MEDLINE, and CENTRAL with additional searches of 2019–2021 annual ASCO and ESMO conferences was conducted to identify studies in the target population. A random-effects meta-analysis was performed among studies involving chemotherapies and targeted therapies relevant in treatment guidelines and HTA activities. Outcomes of interest were objective response rate (ORR), overall survival (OS), and progression-free survival (PFS) presented as Kaplan–Meier data. Randomized controlled trials reporting any of the outcomes of interest were included. For OS and PFS, individual patient-level data were reconstructed from published Kaplan–Meier curves.

**Results:**

Forty-four trials were eligible for the analysis. Pooled ORR (42 trials; 77 treatment arms; 7256 participants) was 15.0% (95% confidence interval (CI) 12.7–17.5%). Median OS from the pooled analysis (34 trials; 64 treatment arms; 60,350 person-months) was 7.9 months (95% CI 7.4–8.5). Median PFS from the pooled analysis (32 trials; 61 treatment arms; 28,860 person-months) was 3.5 months (95% CI 3.2–3.7).

**Conclusion:**

Our study confirms poor prognosis among patients with advanced gastric cancer, following disease progression on first-line therapy. Despite the approved, recommended, and experimental systemic treatments available, there is still an unmet need for novel interventions for this indication.

**Supplementary Information:**

The online version contains supplementary material available at 10.1007/s12029-023-00932-5.

## Introduction

With over a million new cases per year worldwide, gastric cancer is one of the leading causes of cancer-related mortality [1]. Despite declining incidence rates, gastric cancer is the most frequently diagnosed cancer in men in many South Central Asian countries, with the highest incidence rates in Eastern Asia and Eastern Europe [1]. Five-year survival rates range from 23 to 45% in patients with the locally advanced disease who receive chemotherapy before and after surgery [2–4], whereas that of distant metastatic disease is only 6% [5]. With the exception of Asian countries, where routine screening is performed, patients are often diagnosed at a late stage, where surgery is not an option, or with distant metastasis. For those who are diagnosed at earlier stages, surgery is a possible cure; however, the majority will still experience disease progression following resection [6].

Several single-agent and combination therapies have been recommended for advanced gastric cancer, including chemotherapy, chemoradiation, immunotherapy, and targeted therapy, depending on the disease characteristics [6, 7]. Specifically, doublet or triplet chemotherapy regimens containing platinum, fluoropyrimidine, or taxanes are recommended for first-line treatment of advanced gastric cancer [6]. In this setting, patients with HER-2 overexpression positive tumors would benefit from trastuzumab, a HER-2-targeted antibody, in combination with chemotherapy regimens with or without pembrolizumab (an inhibitor of programmed cell death protein 1 (PD-1)) [6, 7]. Patients with HER-2 overexpression negative tumors but with programmed death ligand 1 (PD-L1) combined positive score (CPS) of 5 or above are recommended to receive nivolumab (another PD-1 inhibitor) in combination with chemotherapy [7]. For patients with adequate performance status who experience disease progression on first-line treatments, certain chemotherapy regimens have been shown to improve survival in the second and later lines of treatment when compared to best supportive care [6, 7]. Targeted therapies, such as ramucirumab (an inhibitor of vascular endothelial growth factor (VEGF)), are also recommended for these patients [7]. Despite this, around half of these patients will die within a year [8–18], indicating poor prognosis with the available treatments.

The comparative efficacy of interventions in the second and third lines of treatment for patients with advanced gastric cancer has previously been summarized [19–22]. The objective of this study was to synthesize the clinical prognosis of all-comer patients with pretreated advanced gastric cancer receiving relevant chemo- and targeted therapies (from here on referred to as the target population) through a meta-analysis of relevant randomized controlled trials (RCTs).

## Methods

### Study Identification

A comprehensive systematic literature review was conducted on July 6, 2021, in accordance with the Preferred Reporting Items for Systematic Reviews and Meta-analysis (PRISMA) guidelines [23]. Two reviewers independently performed title/abstract screening, full-text screening, and data extraction of the included studies. At each stage, any discrepancies between reviewers were reconciled through discussion, with a third reviewer being involved to reach consensus on the remaining disagreements. The quality of the trials included in the meta-analysis was evaluated using the Cochrane Collaboration risk of bias assessment tool for RCTs [24]. Of note, the review protocol of this study was not registered with PROSPERO. We searched Embase, MEDLINE, CENTRAL, recent (2019–2021) conference proceedings, and the US clinical trials registry to identify clinical trials conducted in the target population and published since 2000. Search strategies for the systematic review are provided in Online Resource 1.

Population of interest was all-comer patients (i.e., regardless of specific biomarkers) with disease progression on or after at least one prior line of systemic therapy for advanced (unresectable and/or metastatic) gastric cancer. Interventions of interest were systemic therapies that were granted regulatory approval for any indication. Detailed study eligibility criteria of the systematic literature review are presented in Table 1.

**Table 1:** Study eligibility criteria for the systematic literature review and meta-analysis

	**Systematic literature review**	**Meta-analysis (additional criteria only)**
**Criteria**	**Description**	
**Population**	• Adult (≥ 18 years) patients with gastric cancer who previously received systemic therapy for advanced (defined as unresectable and/or metastatic) disease. Recurrent disease was considered advanced stage where resectability and stage were not specified.	
	• Performance status of 0–1 (or equivalent)	
**Interventions**	• Any pharmacologic treatment licensed by the FDA or EMA for any indication (including off-label treatments)	• Studies evaluating at least one of the following treatments: ◾ Folinic acid + 5-FU + oxaliplatin (FOLFOX) ◾ Folinic acid + 5-FU + irinotecan (FOLFIRI) ◾ Ramucirumab + paclitaxel ◾ Docetaxel ◾ Paclitaxel ◾ Carboplatin + paclitaxel ◾ Ramucirumab + docetaxel ◾ Capecitabine ◾ Irinotecan ◾ Trifluridine + tipiracil ◾ Ramucirumab ◾ Carboplatin + irinotecan ◾ Folinic acid + 5-FU + irinotecan + oxaliplatin (FOLFIRINOX) ◾ Epirubicin + cisplatin + capecitabine (ECX) ◾ Epirubicin + oxaliplatin + capecitabine (EOX) ◾ Gemcitabine ◾ 5-FU + carboplatin + docetaxel ◾ 5-FU ◾ Cisplatin ◾ Docetaxel ◾ 5-FU ◾ Apatinib
**Comparators**	• Unrestricted	
**Outcomes**	At least one of the following outcomes: • Objective response rate, disease control rate, and number of patients with complete response, partial response, stable disease, or progressive disease when available • Overall survival • Progression-free survival • Time to progression • Duration of response • Any-cause and treatment-related adverse events (AEs) • Any-cause and treatment-related grade 3–5 AEs • Any-cause and treatment-related serious AEs • Discontinuation due to AEs • Patient-reported outcomes (e.g., EQ-5D, EORTC QLQ-C30)	• Objective response rate • Overall survival as Kaplan–Meier data • Progression-free survival as Kaplan–Meier data
**Study design**	• Randomized controlled trials • Non-randomized trials • Single-arm trials	• Randomized controlled trials
**Time**	• January 1, 2000–July 6, 2021	
**Language**	• English language	

*5-FU* fluorouracil, *AE* adverse event, *EMA* European Medicines Agency, *FDA* Food and Drug Administration

### Meta-Analysis

#### Overview

A meta-analysis was conducted including studies involving chemotherapies and/or targeted therapies relevant in treatment guidelines and HTA activities, as these classes of therapies are standard in treating the target population. Relevant therapies were as follows: folinic acid + 5-FU + oxaliplatin (FOLFOX), folinic acid + 5-FU + irinotecan (FOLFIRI), ramucirumab + paclitaxel, docetaxel, paclitaxel, carboplatin + paclitaxel, ramucirumab + docetaxel, capecitabine, irinotecan, trifluridine + tipiracil, ramucirumab, carboplatin + irinotecan, folinic acid + 5-FU + irinotecan + oxaliplatin (FOLFIRINOX), epirubicin + cisplatin + capecitabine (ECX), epirubicin + oxaliplatin + capecitabine (EOX), gemcitabine, 5-FU + carboplatin + docetaxel, 5-FU, cisplatin, docetaxel, 5-FU, and apatinib.

The primary outcome of interest was the objective response rate (ORR). Secondary outcomes were overall survival (OS) and progression-free survival (PFS) presented as Kaplan–Meier data. Due to the vast amount of literature identified in the systematic review, the study designs of interest for the analysis were limited to RCTs, as this type of trial design provides the highest quality of experimental evidence [25]. RCTs evaluating at least one of the outcomes of interest were eligible for inclusion, and data from all their treatment arms were incorporated into the analysis. Detailed study eligibility criteria of the meta-analysis are presented in Table 1.

Meta-analyses were performed to combine the results from multiple studies in an effort to obtain a precise estimate of the overall rate and/or to resolve uncertainty around the efficacy of treatments for target patients.

Due to inherent differences among the included trials, targeted treatments, dose intensities, study design, length of follow-up, populations, and outcome measurements, heterogeneity was expected. Therefore, a random-effects meta-analysis was used to synthesize the overall estimate of interest, although a fixed-effect model was also planned for in the case of a low number of included treatment arms. Post hoc independent subgroup analyses by therapy class were conducted in the case of high heterogeneity. To assess for potential publication bias, visual inspection of a funnel plot and Egger’s test were performed. Additional details on the statistical methods can be found in Online Resource 1.

#### Objective Response Rate

ORR is defined as the proportion of patients who achieved either complete response or partial response following treatment (i.e., number of responders/total participants). When performing meta-analyses of proportions, it is usually advantageous to first transform the proportions into a measure that has better statistical properties, particularly when the number of events is very small or zero. The Freeman-Tukey double arcsine transformation [26] was used to transform raw response proportion estimates so that the data follow an approximate normal distribution. This approach is recommended as it does not require any adjustments or continuity corrections to the observed data. The model estimate was then back-transformed such that the final estimate was on the original scale (proportion) [27]. Clopper-Pearson 95% confidence intervals (CIs) were computed for individual treatment arms.

#### Time-To-Event Survival Outcomes

Overall survival was defined as the time from the date of first dose to death by any cause. Progression-free survival was defined as the time from the date of first dose to disease progression or death by any cause, whichever occurs first. When summarizing OS and PFS, individual patient-level data were reconstructed from published Kaplan–Meier curves, using the Guyot algorithm [28] and DigitizeIt software version 2.3.3 (www.digitizeit.xyz), and pooling of these curves was performed. This approach enabled patient-level data from studies that only reported aggregated study-level data to be incorporated, allowing the use of time-dependent survival models in the meta-analysis. For the pooling of the reconstructed data across grouped studies, the conditional survival probabilities at each timepoint were arcsine transformed and modeled with a random effect meta-analysis method described by Combescure et al. [29] with study as the random term. The summary survival probabilities were obtained by the product of the pooled conditional survival probabilities. The mean and median survival times were derived from the summary survival curve assuming a linear interpolation of the survival between the points.

#### Software

SAS software version 9.4 [30] was used for conducting the meta-analyses of proportion outcomes R version 4.0.1 [31] was used for the generation of supporting forest plots. Pooling of survival curves was estimated using the *metasurv* package with R version 4.0.1. [31].

#### Presentation of Results

Results presented from the meta-analyses of ORR include fixed-effect and random-effects (pooled) estimates of ORR (with 95% CIs), ${I}^{2}$, ${\tau }^{2}$ statistics, *p*-value for Cochran’s *Q* test for testing heterogeneity, and corresponding forest plots. The ${I}^{2}$ statistic measures the percentage of variation across studies attributed to the heterogeneity among trials rather than chance. Results for each analyzed time-to-event endpoint include the total sample size, number of events, pooled survival curve, overall median survival time with the corresponding 95% CI, ${I}^{2}$ statistic, and *p*-value for Cochran’s *Q* test for testing heterogeneity.

## Results

### Evidence Base

Searches from the main databases, conference proceedings, and other sources resulted in 14,148 records. After removing duplicate records (*n* = 4271) and excluding citations during title/abstract screening (*n* = 8908), 969 citations were advanced to the full-text screening stage. Of these, a total of 265 citations representing 214 unique clinical trials were included in the systematic literature review.

**Fig. 1:**
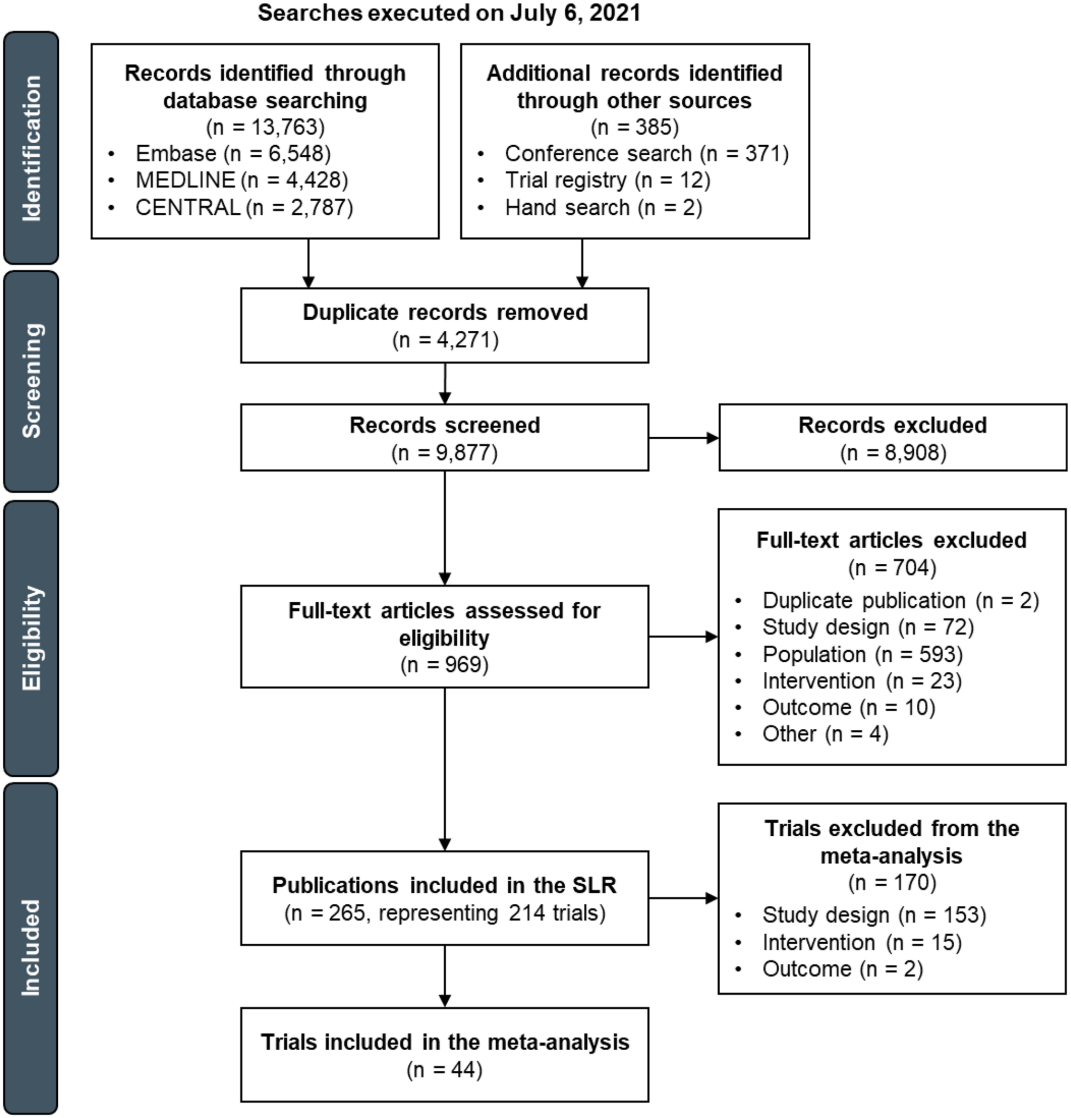
Study selection flow diagram for the systematic review and the meta-analysis. Abbreviations: SLR, systematic literature review

**Fig. 2:**
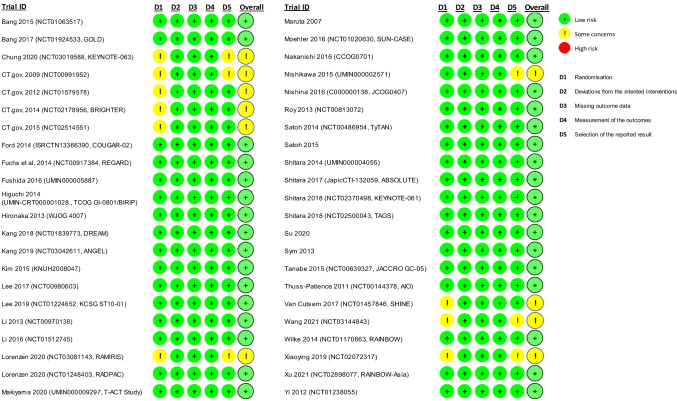
Quality assessment of the included trials

Of the 214 clinical trials included in the systematic literature review, 44 RCTs met the study eligibility criteria of the meta-analysis and were ultimately included (Fig. 1). Around half of the studies (*n* = 21 trials) were in phase II and the other half (*n* = 20) were in phase III; one was a phase II/III trial and two did not report the study phase. Twenty-six RCTs were open-label in design, with 15 being double-, triple-, or quadruple-blinded; three trials did not report on masking. Most of the studies (*n* = 40) were conducted in multiple centers. Thirty-five trials were published as full-text articles, five were published as conference abstracts, and four were only reported in the clinical trials registry. Ten trials were conducted globally and four were from Asia, with 11, seven, and five being exclusively conducted in Japan, Korea, and China, respectively; the remaining studies were conducted in Germany (*n* = 4), Japan and Korea (*n* = 1), the USA (*n* = 1), and the UK (*n* = 1). Trials often had low risk of bias in all evaluated dimensions and were of high quality (Fig. 2). Studies with “some concerns” regarding risk of bias were often only reported in conference abstracts or the clinical trials registry, with minimal information available on the randomization procedure.

Trial sample sizes ranged from 19 to 726 patients, with the proportion of males ranging from 57.1 to 81.9%. Mean age (and median age when mean was not reported) ranged from 52 to 68 years. Where reported, percentage of patients with Eastern Cooperative Oncology Group performance scores of either 0 or 1 ranged from 72.4 to 100%. Lastly, 36 trials were conducted in populations with exactly one prior line of treatment, whereas five trials were conducted in those with ≥ 2 prior lines; the remaining studies recruited patients with at least one prior line of treatment (Table 2).

**Table 2:** Baseline patient characteristics of the treatment arms included in the meta-analysis

**Study (registry code, trial name)**	**Treatment arm**	***N***	**Age, median (range) or mean (SD)**	**Males, *n* (%)**	**ECOG 0 or 1, %**	**Prior lines of therapy, %**	
						**1**	≥** 2**
Bang et al. (2015) [32] (NCT01063517)	Paclitaxel	62	61 (25–79)	44 (71)	96.8	100	0
	Olaparib + paclitaxel	62	63 (31–77)	49 (79)	100	100	0
Bang et al. (2017) [33] (NCT01924533, GOLD)	Paclitaxel	262	59 (50–62)	185 (71)	99.6	100	0
	Olaparib + paclitaxel	263	58 (49–67)	174 (66)	99.6	100	0
Chung (2020) [35] (NCT03019588, KEYNOTE-063)	Paclitaxel	47	--	--	100	100	0
CT.gov (2009) [48] (NCT00991952)	Irinotecan + alvocidib	13	62.5 (19.1)	7 (54)	--	100	0
	Irinotecan	6	56 (17)	6 (100)	--	100	0
CT.gov (2012) [49] (NCT01579578)	AZD8931 + paclitaxel	13	57.9 (8.7)	9 (69)	100	100	0
	Placebo + paclitaxel	12	62.7 (13.6)	9 (75)	100	100	0
CT.gov (2014) [50] (NCT02178956, BRIGHTER)	Placebo + Paclitaxel	357	59.9 (11.1)	254 (71)	100	100	0
	Napabucasin + paclitaxel	357	60.8 (11.5)	261 (73)	100	100	0
CT.gov (2015) [51] (NCT02514551)	Ramucirumab (12) + paclitaxel (80)	40	58.5 (14)	83 (67)	100	100	0
	Ramucirumab (8) + paclitaxel (80)	42	58.7 (10.4)	78 (64)	100	100	0
Ford et al. (2014) [9] (ISRCTN13366390, COUGAR-02)	Docetaxel + active symptom control	84	65 (28–84)	69 (82)	83.3	100	0
Fuchs et al. [16] (NCT00917384, REGARD)	Ramucirumab	238	60 (52–67)	169 (71)	100	100	0
Fushida et al. [68] (UMIN000005887)	Paclitaxel	33	68 (49–84)	--	84.8	67	33
	Paclitaxel + valproic acid	33	67 (31–83)	--	87.9	61	39
Higuchi et al. (2014) [15] (UMIN-CRT000001028., TCOG GI-0801/BIRIP)	Irinotecan	63	67 (49–78)	55 (87)	100	100	0
	Irinotecan + cisplatin	64	66 (29–80)	49 (77)	100	100	0
Hironaka et al. (2013) [14] (WJOG 4007)	Paclitaxel	108	65 (37–75)	84 (78)	96.3	100	0
	Irinotecan	111	65 (38–75)	87 (78)	96.4	100	0
Kang et al. (2018) [37] (NCT01839773, DREAM)	DHP107 (oral paclitaxel)	118	59 (33–83)	91 (77)	98.3	100	0
	Paclitaxel (IV)	118	59 (27–80)	94 (80)	99.2	100	0
Kang et al. (2019) [36] (NCT03042611, ANGEL)	Apatinib + BSC	308	60 (21–91)^a^	241 (78)	100	0	100
Kim et al. (2015) [38] (KNUH2008047)	Docetaxel	27	--	24 (89)	96.3	100	0
	Docetaxel + oxaliplatin	25	--	18 (72)	96	100	0
Lee et al. (2017) [39] (NCT00980603)	Docetaxel	23	56 (34–68)	18 (78)	100	100	0
	Docetaxel + cisplatin	23	55 (38–74)	20 (87)	91.3	100	0
	Docetaxel + S-1	23	55 (39–68)	14 (61)	91.3	100	0
Lee et al. (2019) [40] (NCT01224652, KCSG ST10-01)	Paclitaxel	54	59 (38–82)	38 (70)	96.3	100	0
	Irinotecan	58	59 (38–77)	40 (69)	96.6	100	0
Li et al. (2013) [41] (NCT00970138)	Apatinib 850 QD	47	--	39 (83)	100	0	100
	Apatinib 425 BID	46	--	34 (74)	100	0	100
Li et al. (2016) [42] (NCT01512745)	Apatinib	176	58 (23–71)	132 (75)	100	0	100
Lorenzen et al. (2020b) [61] (NCT03081143 RAMIRIS)	Ramucirumab + paclitaxel	38	58	27 (71)	100	100	0
	Ramucirumab + FOLFIRI	72	61	47 (65)	100	100	0
Lorenzen et al. (2020a) [43] (NCT01248403, RADPAC)	Paclitaxel + placebo	150	62 (29–86)	121 (81)	90	53	47
	Everolimus + paclitaxel	150	62 (32–83)	110 (73)	91.3	62	38
Makiyama et al. (2020) [44] (UMIN000009297, T-ACT Study)	Paclitaxel	45	67 (33–81)	39 (87)	95.6	100	0
	Trastuzumab + paclitaxel	44	65 (50–89)	32 (73)	95.5	100	0
Maruta et al. (2007) [45]	Docetaxel	12	64.8 (4.1)	9 (75)	91.7	100	0
	Docetaxel + 5’dFUR	12	61.3 (10.6)	9 (75)	91.7	100	0
Moehler et al. (2016) [46] (NCT01020630, SUN-CASE)	FOLFIRI + sunitinib	45	62 (37–76)	33 (73)	--	76	22
	FOLFIRI + placebo	45	57 (28–84)	30 (67)	--	76	24
Nakanishi (2016) [47] (CCOG0701)	Paclitaxel	40	62 (38–80)	34 (85)	92.5	100	0
	S1 + paclitaxel	38	64 (42–79)	29 (76)	97.4	100	0
Nishikawa et al. (2015) [52] (UMIN000002571)	Irinotecan + cisplatin	84	67 (36–85)	68 (81)	100	100	0
	Irinotecan	84	68 (35–87)	63 (75)	100	100	0
Nishina et al. (2016) [69] (C000000138, JCOG0407)	5-FU regimen	49	59 (30–74)	33 (67)	98	100	0
	Paclitaxel	51	64 (39–75)	36 (71)	96.1	100	0
Roy et al. (2013) [53] (NCT00813072)	PEP02	44	56 (38–81)	35 (80)	93.2	100	0
	Irinotecan	44	62 (33–79)	34 (77)	93.2	100	0
	Docetaxel	44	58 (33–81)	34 (77)	90.9	100	0
Satoh et al. (2014) [18] (NCT00486954, TyTAN)	Paclitaxel	129	62 (22–80)	106 (82)	100	100	0
	Lapatinib + paclitaxel	132	61 (32–79)	101 (77)	100	100	0
Satoh et al. (2015) [54]	Irinotecan	42	64 (32–75)	33 (79)	100	100	0
Shitara et al. (2014) [57] (UMIN000004055)	Paclitaxel (standard-dose weekly)	45	65 (33–80)	29 (64)	91.1	56	44
	Paclitaxel (dose-escalated weekly)	44	62 (29–78)	32 (73)	90.9	55	45
Shitara et al. (2017) [56] (JapicCTI-132059, ABSOLUTE)	Nab-paclitaxel	243	66 (60–72)	178 (73)	98.4	100	0
	Nab-paclitaxel	240	67 (60–72)	178 (74)	99.2	100	0
	Solvent-based paclitaxel	243	65 (59–71)	176 (72)	98.4	100	0
Shitara et al. (2018) [55] (NCT02370498, KEYNOTE-061)	Paclitaxel	296	60 (53–68)	80 (30)	99.7	100	0
Shitara et al. (2018) [55] (NCT02500043, TAGS)	Trifluridine/tipiracil	337	64 (56–70)	252 (75)	100	0	100
Su (2020) [58]	Apatinib	35	62.3 (15)	21 (60)	80	0	100
Sym et al. (2013) [59]	Irinotecan	29	60 (45–76)	20 (69)	93.1	100	0
	mFOLFIRI	30	61 (30–75)	14 (47)	90	100	0
Tanabe et al. (2015) [60] (NCT00639327, JACCRO GC-05)	S-1 + irinotecan	145	67 (37–84)	99 (68)	100	100	0
	Irinotecan	148	66 (22–83)	109 (74)	100	100	0
Thuss-Patience et al. (2011) [62] (NCT00144378, AIO)	Irinotecan	21	58 (43–73)	18 (86)	81	100	0
Van Cutsem et al. (2017) [63] (NCT01457846, SHINE)	AZD4547	41	60.6 (11.4)	29 (71)	--	100	0
	Paclitaxel	30	61.9 (10.7)	22 (73)	--	100	0
Wang et al. (2021) [64] (NCT03144843)	Paclitaxel	23	--	--	100	100	0
	Apatinib + paclitaxel	21	--	--	100	100	0
Wilke et al. (2014) [17] (NCT01170663, RAINBOW)	Ramucirumab + paclitaxel	330	61 (25–83)	229 (69)	100	100	0
	Paclitaxel + placebo	335	61 (24–84)	243 (73)	100	100	0
Xiaoying et al. (2019) [65] (NCT02072317)	Paclitaxel	75	--	(63.5) ^b^	100	100	0
	Paclitaxel + raltitrexed	73	--	(63.5) ^b^	100	100	0
Xu et al. (2021) [66] (NCT02898077, RAINBOW-Asia)	Ramucirumab + paclitaxel	294	57 (22–84)	205 (70)	100	100	0
	Placebo + paclitaxel	146	58 (18–79)	96 (66)	100	100	0
Yi et al. (2012) [67] (NCT01238055)	Docetaxel + sunitinib	56	54 (20–72)	40 (71)	89.3	100	0
	Docetaxel	49	52 (36–70)	33 (67)	93.9	100	0

*SD* standard deviation, *ECOG* Eastern Cooperative Oncology Group performance score

^a^Mean and range were reported

^b^There were 94 males in the entire population. Reported percentages are based on the overall population

Among treatment arms with active interventions, 38 unique regimens were evaluated in the included RCTs. Of these, eight were single-agent chemotherapies, 14 were combination chemotherapies, three were single-agent targeted therapies, nine were combinations of chemo- and targeted therapies, and two were chemotherapy agents in combination with other treatment classes. Lastly, two interventions (pembrolizumab and nimotuzumab + irinotecan) were immunotherapies and were therefore excluded from the meta-analysis.

### Objective Response Rate

**Fig. 3:**
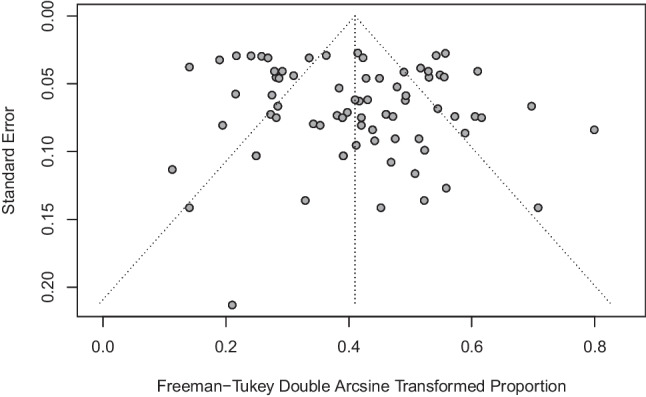
Funnel plot for the assessment of publication bias. Funnel plot of the estimate in the random effects model of 77 trial arms included in the meta-analysis of objective response rate is symmetrical. Accompanying Egger’s test did not show any evidence of publication bias (intercept = 1.06, 95% CI −0.28 to 2.40, *p* = 0.12)

**Fig. 4:**
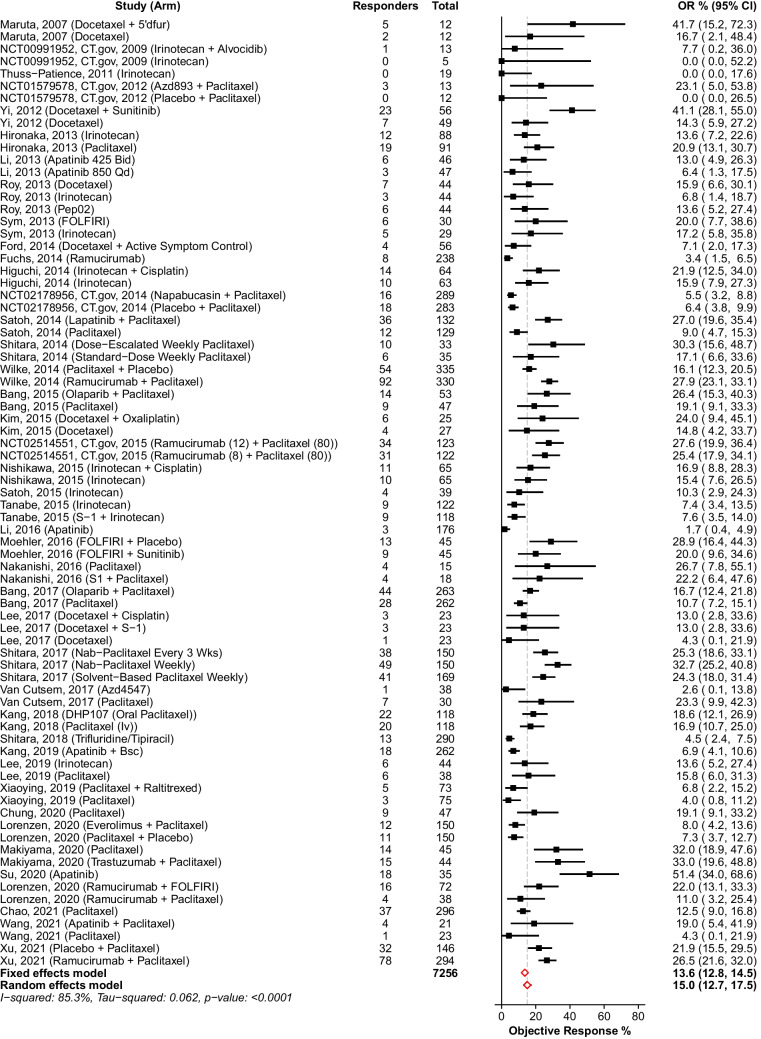
Meta-analysis of objective response rate. Abbreviations: CI, confidence interval; OR, objective response

Seventy-seven treatment arms from 42 RCTs [9, 14–18, 32–67] were included in the analysis. A funnel plot and Egger’s test were performed to assess potential publication bias in the literature (Fig. 3). Both visual inspection of the funnel plot and Egger’s test did not show any evidence of publication bias (intercept = 1.06, 95% CI−0.28 to 2.40, *p* = 0.12). The random effect ORR pooled estimate was 15.0% (95% CI 12.7–17.5%). The corresponding statistics for heterogeneity were ${I}^{2}$ = 85.3%, ${\tau }^{2}$ = 0.062, and $p$ < 0.0001 (Fig. 4). Due to this high level of heterogeneity (*I*^2^ = 85.3%), a post hoc subgroup analysis by therapy class was conducted. Subgroups were defined by single-agent chemotherapies (*n* = 41 trial arms), combination chemotherapies (*n* = 12 trial arms), single-agent targeted therapies (*n* = 7 trial arms), and combination chemo- and targeted therapies (*n* = 17 trial arms). Meta-analyses revealed single-agent targeted therapies exhibited the lowest ORR at 8.6% (95% CI 3.0–16.3%, *I*^2^ = 89.8%), followed by single-agent chemotherapies at 14.0% (95% CI 11.6–16.7, *I*^2^ = 73.7%), combination chemotherapies at 15.5% (95% CI 9.5–22.6%, *I*^2^ = 79.3%), and combination chemo- and targeted therapies with the highest ORR at 20.9% (95% CI 15.7–26.7%, *I*^2^ = 87.4%).

### Overall Survival

**Fig. 5:**
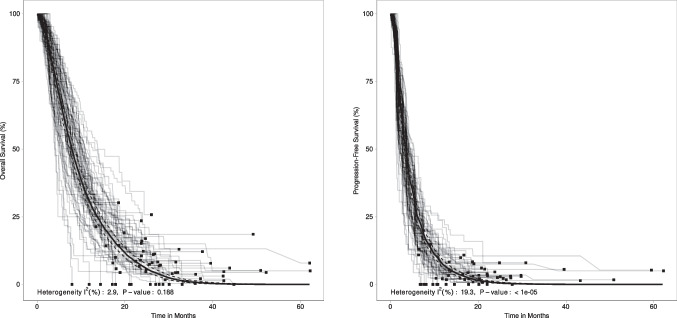
Kaplan–Meier estimates of overall survival and progression-free survival. The grey lines represent the Kaplan–Meier estimates for survival events in each treatment arm. The black squares represent the end of follow-up for each corresponding treatment arm. The thick black line represents the random effects pooled survival curve estimate with 95% confidence bands (dashed lines). *P*-value refers to Cochran’s Q test for heterogeneity. **A** Kaplan–Meier estimates of overall survival among 61 treatment arms included in the analysis. **B** Kaplan–Meier estimates of progression-free survival among 61 treatment arms included in the analysis

Sixty-four treatment arms from 34 RCTs [9, 14–18, 32–34, 36, 38–41, 43–47, 52–57, 59–61, 62, 63, 66–69] were included in the analysis of overall survival. Median OS from the pooled analysis was 7.9 months (95% CI 7.4–8.5 months). Pooled OS rates at 6, 12, and 24 months were 62.5%, 30.4%, and 6.7%, respectively (Table 3). The corresponding statistics for heterogeneity were ${I}^{2}$ = 2.9% and $p$ = 0.188 (Fig. 5A).

**Table 3:** Meta-analysis of survival outcomes

	**Overall survival**^**a**^	**Progression-free survival**^**b**^
Number of participants	6512	6532
Number of trials	34	32
Number of treatment arms	64	61
Number (%) of events	5,390 (82.8)	5887 (90.1)
Person-months^c^	60,350	28,860
Event rate per 100 person-months	8.9	20.4
Median (95% CI) survival (months)^d^	7.9 (7.4–8.5)	3.5 (3.2–3.7)
Rate at 6 months, % (95% CI)^d^	62.5 (59.0–66.2)	25.8 (23.1–28.8)
Rate at 12 months, % (95% CI)^d^	30.4 (27.7–33.3)	7.3 (6.0–8.9)
Rate at 24 months, % (95% CI)^d^	6.7 (5.4–8.4)	0.7 (0.4–1.0)
Rate at 48 months, % (95% CI)^d^	0.1 (0.0–0.2)	0.0 (0.0–0.0)
*I*^2^ value (heterogeneity)^d^	2.9%	19.3%
p-value for Cochran’s *Q* test^d^	0.188	< 0.00001

*CI* confidence interval

^a^Overall survival was defined as time from date of first dose to death by any cause

^b^Progression-free survival was defined as time from date of first dose to disease progression or death by any cause, whichever occurs first

^c^Calculated as the total time-at-risk (in months) for all individuals across all treatment arms

^d^Estimates derived from the random effects pooled survival curve using the methodology from Combescure et al. [29]

### Progression-Free Survival

Sixty-one treatment arms from 32 RCTs [14–18, 32–34, 36–41, 43, 44, 46, 47, 52–61, 63, 66, 68, 69] were included in the analysis of progression-free survival. Median PFS from the pooled analysis was 3.5 months (95% CI 3.2–3.7 months). Pooled PFS rates at 6, 12, and 24 months were 25.8%, 7.3%, and 0.7%, respectively (Table 3). The corresponding statistics for heterogeneity were ${I}^{2}$ = 19.3% and $p$ < 0.00001 (Fig. 5B).

## Discussion

We aimed to synthesize and summarize all available relevant clinical evidence on the absolute efficacy of second- or later-line systemic therapies in patients with advanced gastric cancer to inform clinicians, patients, and healthcare decision makers. The target population included all those receiving a second or later line of treatment for advanced gastric cancer, regardless of the specific biomarkers that can guide the effective treatment [70], (i.e., HER-2 overexpression, high levels of microsatellite instability (MSI-H), mismatch repair deficiency (dMMR), programmed death ligand 1 (PD-L1) overexpression, and fibroblast growth factor receptor (FGFR) alterations). Outcomes of interest were ORR, OS, and PFS, as these were deemed the key endpoints in clinical trials of advanced gastric cancer.

Data from 7256 patients were incorporated in the analysis of ORR, with a total time-at-risk of 60,350, and 28,860 person-months for the analyses of OS and PFS, respectively. The estimated pooled ORR was 15.0%, and the median OS and PFS were 7.9 months and 3.5 months, respectively. To our knowledge, the current analysis is the only study that has quantitatively synthesized the clinical prognosis of patients treated with relevant chemo- and targeted therapies in the post-first-line setting. This is important because our findings indicate that clinical outcomes are still poor in the target population despite certain interventions being more efficacious than others, as shown in previously published analyses [20, 22, 71]. Furthermore, this research could be of value to many stakeholders including health technology assessment bodies, policy makers, physicians, patients, clinical development program scientists, and researchers. First, health technology assessment bodies and policy makers could use these results as a benchmark to contextualize the efficacy of novel treatments in the second- or later-line setting. Second, physicians could use these efficacy estimates in communicating with patients as well as in the medical decision-making process. Third, these results could be used in clinical development programs for defining hypotheses and statistical power calculations. Fourth, this research uses a state-of-the-art method of performing a meta-analysis on published, and then digitized survival curves. Researchers could further apply this technique to future applications in gastrointestinal cancer and other cancers.

Inference from a systematic review and meta-analysis approach has some limitations: the potential for publication bias should be noted although we performed hand searches of recent conference proceedings and clinical trial registry results to capture any available outcome data that may not have been published. In addition, a funnel plot and Egger’s test did not indicate any potential publication bias. Differences in observation periods could cause biased estimates for certain outcomes or treatments. Furthermore, although interventions recommended by clinical practice guidelines and relevant to health technology assessment submissions were included in the analyses, therapies used in routine clinical practice and/or approved in specific regions may not have been included. Immunotherapies were excluded from analyses as these therapies are currently only recommended in certain circumstances (e.g., microsatellite instability high (MSI-H), deficient mismatch repair (dMMR), high tumor mutation burden (TMB-high) tumors) and are not yet recommended for the broader, all-comers, pretreated population (NCCN Guidelines Version 2.2022). In addition, participants in the included studies are, for the vast majority, recorded before the introduction of these novel therapeutics. Lastly, there was heterogeneity among the studies in terms of phase of the trials, sample size, the region studies were conducted in, level of masking, and patient characteristics. Specifically, populations varied in terms of the number of prior lines of treatment, which may have affected the efficacy of the evaluated interventions. Since the results of individual trials depend on the trial design and participant characteristics, in the absence of individual patient-level data, potential underlying differences across the included trials (including the above-mentioned variables) could not be adjusted for beyond modelling with random effect meta-analysis. Note that the observed treatment effects across individual trials were less heterogeneous in terms of OS (${I}^{2}$ = 2.9%) and PFS (${I}^{2}$ = 19.3%) compared to ORR (${I}^{2}$ = 85.3%).

Despite these limitations, the trials included in our analysis were identified based on a rigorous and comprehensive systematic review, which searched the published literature as well as recent conference proceedings and the US clinical trial registry based on pre-specified eligibility criteria. The entire systematic review was conducted by two reviewers, following PRISMA guidelines to ensure the accuracy and robustness of findings. For the analysis of survival outcomes, we not only used published OS and PFS rates, but also leveraged the information retrieved via digitizing the published Kaplan–Meier figures, thereby allowing the use of time-dependent survival models in the meta-analysis. The statistical approaches used in the meta-analysis were previously established in the published literature, and both fixed effect and random effects estimates were evaluated.

Overall, our findings are consistent with the current understanding of the efficacy of conventional chemotherapy and targeted therapies for pretreated patients with advanced gastric cancer, emphasizing that prognosis remains poor in those who receive them as salvage regimens [72]. Beyond these conventional treatments, however, various immunotherapies are approved, recommended, or being investigated in patients with advanced gastric cancer [7, 73]. Monoclonal antibodies against PD-1, its ligand (PD-L1), or cytotoxic T lymphocyte-associated antigen 4 (CTLA-4) can be administered as monotherapy or in combination with chemotherapy, targeted therapy (e.g., anti-HER-2 therapy, inhibitors of VEGF or its receptor (VEGFR)), or another immunotherapy [73, 74]. Specifically, patients with MSI-H/dMMR tumors and those with TMB-high are recommended to receive pembrolizumab, a PD-1 inhibitor, as second-line or subsequent therapy [7].

Our findings confirm poor prognosis among patients with advanced gastric cancer, with disease progression on first-line therapy. Payers, physicians, and patients could use these findings to contextualize the efficacy of novel therapies. Despite the approved, recommended, and experimental systemic treatments available, there is still an unmet need novel interventions for advanced gastric cancer patients receiving second or later lines of treatment.

### Supplementary Information

Below is the link to the electronic supplementary material.

Supplementary file1 (PDF 282 KB)

## Data Availability

The datasets analyzed during the current study were generated based on the data published in the citations included in this manuscript.
