# Supplementary material for: Clinical Outcomes for Previously Treated Patients with Advanced Gastric or Gastroesophageal Junction Cancer: A Systematic Literature Review and Meta-Analysis
Source: J Gastrointest Cancer. 2023 May 23;54(4):1031–45. doi: 10.1007/s12029-023-00932-5 (PMC10754747; doi:10.1007/s12029-023-00932-5)
Supplement: Supplementary file 1 — Supplementary file1 (PDF 282 KB) [file 12029_2023_932_MOESM1_ESM.pdf]

# Clinical outcomes for previously-treated patients with advanced gastric or gastroesophageal junction cancer: a systematic literature review and meta-analysis

## Supplementary Appendix

**Journal of Gastrointestinal Cancer**

**Authors:** Lauren A. Abderhalden[1], Ping Wu[2], Mayur M. Amonkar[3], Brian M. Lang[1], Sukrut Shah[3], Fan Jin[3], Andrew M. Frederickson[4], Ali Mojebi[2]

[1]MSD, Zurich, Switzerland

[2]PRECISIONheor, Vancouver, BC, Canada

[3]Merck & Co., Inc., Rahway NJ, USA

[4]PRECISIONheor, New York, NY, USA

**Corresponding author:**

Lauren A. Abderhalden

MSD, Zurich, Switzerland

lauren.abderhalden@msd.com

**Caption**

This supplementary appendix provides the search strategies for the systematic literature review and additional details on the statistical methods used in the meta-analysis.

# Appendix A: Systematic literature review search strategies

## Table A1: Search strategy for Embase

## Embase 1974 to 2021 July 2; Search executed: 6 July 2021

| No. | Terms | Hits |
|---|---|---|
| 1 | exp stomach carcinoma/ | 36,201 |
| 2 | exp stomach cancer/ | 128,756 |
| 3 | exp stomach adenocarcinoma/ | 15,485 |
| 4 | ((stomach or gastric) adj3 (cancer* or carcinoma* or tumo?r* or neoplasm*)).ti,ab. | 129,319 |
| 5 | ((stomach or gastric) adj3 adenocarcinoma*).ti,ab. | 15,694 |
| 6 | ((gastroesophageal or esophagogastric) adj3 (cancer* or carcinoma* or tumo?r* or neoplasm*)).ti,ab. | 3,618 |
| 7 | ((gastroesophageal or esophagogastric) adj3 adenocarcinoma*).ti,ab. | 2,645 |
| 8 | (advance$ or metasta$ or recurr$ or unresect$ or non-resect$ or disseminated or stage 3 or stage III* or stage 4 or stage IV* or spread$ or migration$ or progress$ or invasive or aggressive or "not operable" or untreatable or "not treatable" or secondary or incurable or "not curable").mp. | 6,262,332 |
| 9 | or/1-7 and 8 | 87,549 |
| 10 | Clinical Trial/ | 1,006,558 |
| 11 | Randomized Controlled Trial/ | 664,630 |
| 12 | controlled clinical trial/ | 463,655 |
| 13 | multicenter study/ | 292,184 |
| 14 | Phase 3 clinical trial/ | 54,085 |
| 15 | Phase 4 clinical trial/ | 4,354 |
| 16 | exp RANDOMIZATION/ | 91,387 |
| 17 | Single Blind Procedure/ | 43,058 |
| 18 | Double Blind Procedure/ | 185,378 |
| 19 | Crossover Procedure/ | 67,415 |
| 20 | Placebo/ | 368,060 |
| 21 | randomi?ed controlled trial$.tw. | 261,138 |
| 22 | rct.tw. | 42,528 |
| 23 | (random$ adj2 allocat$).tw. | 47,044 |
| 24 | single blind$.tw. | 27,191 |
| 25 | double blind$.tw. | 221,169 |
| 26 | ((treble or triple) adj blind$).tw. | 1,394 |
| 27 | Placebo$.tw. | 327,885 |
| 28 | Prospective Study/ | 694,714 |
| 29 | (single arm).tw. | 19,411 |
| 30 | (Phase II or Phase 2).tw. | 138,285 |
| 31 | Phase 2 clinical trial/ | 88,908 |
| 32 | or/10-31 | 2,603,096 |
| 33 | Case Study/ | 79,297 |
| 34 | case report.tw. | 453,197 |
| 35 | abstract report/ or letter/ | 1,201,639 |
| 36 | Conference proceeding.pt. | - |
| 37 | Conference abstract.pt. | 4,120,649 |
| 38 | Editorial.pt. | 695,309 |
| 39 | Letter.pt. | 1,179,771 |
| 40 | Note.pt. | 855,878 |
| 41 | or/33-40 | 7,335,062 |
| 42 | 32 not 41 | 1,900,510 |
| 43 | 9 and 42 | 8,314 |
| 44 | limit 43 to yr = 2000 - current | 7,162 |
| 45 | limit 44 to english | 6,548 |

## Table A2: Search strategy for MEDLINE

## Ovid MEDLINE In-Process & Other Non-Indexed Citations, Ovid MEDLINE(R) Daily and Ovid MEDLINE(R) 1946 to July 2, 2021; Search executed: 6 July 2021

| No. | Terms | Hits |
|---|---|---|
| 1 | exp Stomach Neoplasms/ | 100,464 |
| 2 | ((stomach or gastric) adj3 (cancer* or carcinoma* or tumo?r* or neoplasm*)).ti,ab. | 98,540 |

| No. | Terms | Hits |
| --- | --- | --- |
| 3 | ((stomach or gastric) adj3 adenocarcinoma*).ti,ab. | 10,987 |
| 4 | ((gastroesophageal or esophagogastric) adj3 (cancer* or carcinoma* or tumo?r* or neoplasm*)).ti,ab. | 2,147 |
| 5 | ((gastroesophageal or esophagogastric) adj3 adenocarcinoma*).ti,ab. | 1,545 |
| 6 | (advance$ or metasta$ or recurr$ or unresect$ or non-resect$ or disseminated or stage 3 or stage III* or stage 4 or stage IV* or spread$ or migration$ or progress$ or invasive or aggressive or "not operable" or untreatable or "not treatable" or secondary or incurable or "not curable").mp. | 4,583,437 |
| 7 | or/1-5 and 6 | 58,517 |
| 8 | Randomized Controlled Trials as Topic/ | 145,936 |
| 9 | randomized controlled trial/ | 536,565 |
| 10 | Random Allocation/ | 105,549 |
| 11 | Double Blind Method/ | 165,606 |
| 12 | Single Blind Method/ | 30,458 |
| 13 | clinical trial/ | 529,679 |
| 14 | clinical trial, phase i.pt | 21,894 |
| 15 | clinical trial, phase ii.pt | 35,251 |
| 16 | clinical trial, phase iii.pt | 18,640 |
| 17 | clinical trial, phase iv.pt | 2,129 |
| 18 | controlled clinical trial.pt | 94,267 |
| 19 | randomized controlled trial.pt | 536,565 |
| 20 | multicenter study.pt | 298,371 |
| 21 | clinical trial.pt | 529,679 |
| 22 | exp Clinical Trials as topic/ | 360,211 |
| 23 | or/8-22 | 1,441,836 |
| 24 | (clinical adj trial$).tw | 403,563 |
| 25 | ((singl$ or doubl$ or treb$ or tripl$) adj (blind$3 or mask$3)).tw | 181,215 |
| 26 | PlaceboS/ | 35,560 |
| 27 | Placebo$.tw | 226,500 |
| 28 | randomly allocated.tw | 31,324 |
| 29 | (allocated adj2 random$).tw | 34,769 |
| 30 | (single arm).tw. | 9,069 |
| 31 | or/24-30 | 691,831 |
| 32 | 23 or 31 | 1,738,409 |
| 33 | case report.tw | 338,123 |
| 34 | letter/ | 1,142,035 |
| 35 | historical article/ | 364,269 |
| 36 | or/33-35 | 1,827,515 |
| 37 | 32 not 36 | 1,698,904 |
| 38 | 7 and 37 | 6,000 |
| 39 | limit 38 to yr = 2000 - current | 4,865 |
| 40 | limit 39 to english | 4,428 |

## Table A3: Search strategy for CENTRAL

## EBM Reviews - Cochrane Central Register of Controlled Trials May 2021; Search executed: 6 July 2021

| No. | Terms | Hits |
| --- | --- | --- |
| 1 | exp Stomach Neoplasms/ | 2,693 |
| 2 | ((stomach or gastric) adj3 (cancer* or carcinoma* or tumo?r* or neoplasm*)).ti,ab. | 7,988 |
| 3 | ((stomach or gastric) adj3 adenocarcinoma*).ti,ab. | 1,381 |
| 4 | ((gastroesophageal or esophagogastric) adj3 (cancer* or carcinoma* or tumo?r* or neoplasm*)).ti,ab. | 582 |
| 5 | ((gastroesophageal or esophagogastric) adj3 adenocarcinoma*).ti,ab. | 648 |
| 6 | (advance$ or metasta$ or recurr$ or unresect$ or non-resect$ or disseminated or stage 3 or stage III* or stage 4 or stage IV* or spread$ or migration$ or progress$ or invasive or aggressive or "not operable" or untreatable or "not treatable" or secondary or incurable or "not curable").mp. | 507,993 |
| 7 | or/1-5 and 6 | 6,086 |
| 8 | limit 7 to yr = 2000 - current | 5,386 |
| 9 | limit 8 to english | 2,787 |

## Appendix B: Additional details on the statistical methods

Meta-analyses were performed to combine the results from multiple studies in an effort to obtain a precise estimate of the overall rate and/or to resolve uncertainty around the efficacy of treatments for patients with advanced gastric cancer who have progressed on prior treatment.

### Objective response rate

*Double arcsine transformation*

The Freeman-Tukey double arcsine methodology used in the transformation of the ORR outcomes is:

$$\boldsymbol{y = g(p) = \arcsin\sqrt{\left(\frac{r}{(n+1)}\right)} + arcsin\sqrt{\left(\frac{(r+1)}{(n+1)}\right)}} \qquad (1)$$

where *y* is the transformed proportion, *g(.)* represents corresponding function to transform proportion *p*, *r* is the number of responders, and *n* is the number of an individuals in the study.[1] The corresponding variance for each individual study is then computed with variance $v = \frac{1}{(\mathrm{n}+0.5)}$ . The synthesized estimate $\hat{y}$ based on a meta-analysis is back-transformed and computed to get $\hat{p}$ using the formula below.

$$\boldsymbol{\hat{p} = \frac{1}{2}\left[1 - sign(\cos(\hat{y}))\sqrt{\left[1 - \left(\sin(\hat{y}) + \frac{\sin(\hat{y}) - \frac{1}{\sin(\hat{y})}}{n}\right)\text{^}2\right]}\right]} \qquad (2)$$

To back transform the average (pooled estimate) $\hat{p}$, a value of sample size is required. Miller suggests using the harmonic mean of the individual sample sizes.[1] The *n* should be replaced by the harmonic mean of the $n_i$ (of individual studies) to compute the estimate.

*Data conventions for objective response rate*

ORR and number of responders were at times not explicitly reported in all publications. For such cases, ORR and number of responders were taken directly from the reported results. If ORR was not directly available, it was computed by adding complete and partial response events. If the number of responders was not reported, this value was estimated from the reported ORR and total number of subjects randomized (N) in the RCT treatment arm. If the complete and partial values were not reported directly, but the number of events of CR and PR was reported, the complete and partial response rates were estimated, as well as ORR. Where a value for partial response was reported but complete response was not reported, it was assumed that the number of complete responders was zero.

### Time-to-Event Outcomes

When summarizing survival curves, Kaplan-Meier survival curves from literature sources were digitized and pooling of these curves was performed. The following sections describe the pooling of extracted survival data and derivation of pooled survival estimates.

### *Survival curve pooling*

For the pooling of extracted Kaplan-Meier curves across grouped studies, we used methods described in Combescure et al.[2] The conditional survival probabilities at each timepoint were arcsine transformed and pooled assuming a fixed effect or random effects. For random effects, the multivariate methodology of DerSimonian and Laird was applied and the between-study covariances were accounted for. The summary survival probabilities were obtained by the product of the pooled conditional survival probabilities. The mean and median survival times were derived from the summary survival curve assuming a linear interpolation of the survival between the points.

The pooled mean survival time can be calculated as the area under the summary survival curve, which is defined by the pooled survival probabilities. The mean survival time μ was estimated as:

$$\boldsymbol{\hat{\mu} = \sum_{j=1}^{J} 0.5\,(t_j - t_{j-1})\,[\hat{S}^{DL}(t_j) + \hat{S}^{DL}(t_{j-1})]} \quad (3)$$

where *j*= 1,…, J is the set of unique timepoints extracted from the all studies, *t* represents unique timepoints, and $\hat{S}^{DL}(.)$ is the estimated survival probability based on DerSimonian and Laird.

Note that this formula assumes that the estimated probability of survival at the final time point is very small, ideally zero, so that the total area under the survival curve is well approximated.

A 95% confidence interval was obtained by transforming the pooled conditional survival probabilities using a multivariate normal distribution. Calculation of the pooled median survival time, denoted by $t_{med}$, was obtained by using a linear interpolation of the summary survival curve between two successive time points. Denoting $t_m$ the time for which $\hat{S}^{DL}(t_m) < 0.5\ or\ \hat{S}^{DL}(t_{m-1}) > 0.5$, the pooled median survival time was calculated as,

$$\boldsymbol{t_{med} = t_m - (t_m - t_{m-1})\frac{\hat{S}^{DL}(t_m) - 0.5}{\hat{S}^{DL}(t_m) - \hat{S}^{DL}(t_{m-1})}} \quad (4)$$

A 95% confidence interval was obtained by the following procedure.

Applying Greenwood's formula, the variance of the logarithm of $\hat{S}^{DL}(t_j)$ was derived from the terms of covariance matrix of $\hat{\pi}^{DL}$ in which it provides

$$Var\left[\log \hat{S}^{DL}(t_j)\right] = 4 \sum_{u \leqslant j} \sum_{v \leqslant j} Cov\left[\log \sin\left(\hat{\pi}_u^{DL}\right); \log \sin\left(\hat{\pi}_v^{DL}\right)\right]$$

Using the delta method, the covariance terms in the above Equation, one can be approximated by:

$$Cov\left[\log\sin\left(\hat{\pi}_u^{DL}\right);\log\sin\left(\hat{\pi}_v^{DL}\right)\right]=\frac{Cov\left(\hat{\pi}_u^{DL};\hat{\pi}_v^{DL}\right)}{\tan\left(\hat{\pi}_u^{DL}\right)\tan\left(\hat{\pi}_v^{DL}\right)}$$

If a fixed effect model was assumed, the covariance between ${\hat{\pi}_u}^{DL}$ and ${\hat{\pi}_v}^{DL}$ was zero for $u \neq v$. The 95% confidence around the pooled survival was thus obtained by taking the exponential of the logarithm of the pooled survival plus or minus 1.96 times the standard error. However, this 95% confidence interval held only if the logarithm of the pooled survival was normally distributed.

When the pooled survival was close to one, this assumption could not be supported, and the 95% confidence interval around the logarithm could contain the value 0, especially if the variance of the pooled estimates $\hat{\pi}^{DL}$ was large. In such circumstances, the 95% confidence interval was obtained by a Monte Carlo procedure:

- Generate $N_b$ independent copies $\pi^*$from a multinormal with mean $\hat{\pi}^{DL}$ and variance $\hat{V}^{DL}$.
- Calculate the pooled survival probabilities corresponding to each copy $\pi^*$.
- Take the $0.025^{th}$ and $0.975^{th}$ quantiles of the pooled survival probabilities.

The 95% confidence interval around the summary survival probability was guaranteed to be in the interval [0, 1].

### *Survival probability*

The selection of timepoints had an impact of the bias of our estimation, with the inclusion of time intervals containing very few events leading to a downward bias in estimated pooled survival. Therefore, for a given meta-analysis, we calculated pooled survival probabilities at every ½ month of follow-up time, until no included treatment arm had participants-at-risk. In this way, we could show both short-term and long-term trends in survival. Confidence intervals were retrieved via Greenwood's formula.

#### *Heterogeneity*

Due to inherent differences among trials selected through the systematic review, heterogeneity among treatments, dose intensities, study design, length of follow-up, populations, and outcome measurements was expected. Therefore, the primary approach for the meta-analysis was to synthesize the overall estimate of interest using the random effects approach. An inverse-variance weighted random effects meta-analysis was carried out using the DerSimonian-Laird moment method to estimate the between-study (heterogeneity) variance ($\tau^2$).[3] Additionally, fixed effect meta-analyses were performed. If less than five treatment arms were included in a meta-analysis, we were going to use the fixed effect meta-analysis

estimate; this was due to the imprecise heterogeneity estimations when using random effects in the case of low numbers of treatment arms.[4]

The $I^2$ statistic measured the percentage of variation across treatment arms that was due to heterogeneity among trials rather than chance. The $\tau^2$ statistics were a DerSimonian-Laird measure of between-trial (heterogeneity) variance. A high value for $I^2$ was indicative of the heterogeneity above chance and it reduced the representativity of the meta-analysis estimate and its use in generalizability beyond the observed sample of trials.

## References


1. Miller JJ. The inverse of the Freeman–Tukey double arcsine transformation. The American Statistician. 1978;32(4):138-.
2. Combescure C, Foucher Y, Jackson D. Meta-analysis of single-arm survival studies: a distribution-free approach for estimating summary survival curves with random effects. Statistics in Medicine. 2014;33(15):2521-37. doi: https://doi.org/10.1002/sim.6111.
3. DerSimonian R, Laird N. Meta-analysis in clinical trials. Controlled clinical trials. 1986;7(3):177-88.
4. IQWiG: General Methods version 6.0. https://www.iqwig.de/methoden/general-methods_version-6-0.pdf?rev=194070 (2020). Accessed May 25, 2022.
